# Enlarged choroid plexus is linked with poorer physical function in rural older adults: a population-based study

**DOI:** 10.1186/s12987-025-00642-z

**Published:** 2025-04-03

**Authors:** Qianqian Xie, Ziwei Chen, Jiafeng Wang, Huisi Zhang, Yan Wang, Xiaoyu Wang, Chunyan Li, Yongxiang Wang, Lin Cong, Daniel Ferreira, Anna-Karin Welmer, Lin Song, Yifeng Du, Chengxuan Qiu

**Affiliations:** 1 Department of Neurology, Shandong Provincial Hospital Affiliated to Shandong First Medical University, Jinan, Shandong P.R. China; 2 Department of Neurology, Shandong Provincial Hospital, Shandong University, Jinan, Shandong P.R. China; 3 Shandong Provincial Clinical Research Center for Neurological Diseases, Jinan, Shandong P.R. China; 4 Institute of Brain Science and Brain-Inspired Research, Shandong First Medical University, Shandong Academy of Medical Sciences, Jinan, Shandong P.R. China; 5 Aging Research Center, Department of Neurobiology, Care Sciences and Society, Karolinska Institutet-Stockholm University, Stockholm, Sweden; 6 Division of Clinical Geriatrics, Center for Alzheimer Research, Department of Neurobiology, Care Sciences and Society, Karolinska Institutet, Stockholm, Sweden; 7 Facultad de Ciencias de la Salud, Universidad Fernando Pessoa Canarias, Las Palmas, Spain; 8 Division of Physiotherapy, Department of Neurobiology, Care Sciences and Society, Karolinska Institutet, Stockholm, Sweden; 9 Medical Unit Medical Psychology, Women´s Health and Allied Health Professionals Theme, Karolinska University Hospital, Stockholm, Sweden

**Keywords:** Choroid plexus, Physical function, Lateral ventricle, White matter hyperintensity, Population-based study

## Abstract

**Background:**

The choroid plexus (ChP) plays an important role in producing cerebrospinal fluid (CSF) and physical dysfunction has been associated with alterations in CSF circulation. However, no population-based studies have thus far examined the association of ChP with physical function in older people.

**Methods:**

This population-based cross-sectional study included 1217 participants (age ≥ 60 years; 57.35% women) in the MRI substudy of the Multimodal Interventions to delay Dementia and disability in rural China. ChP volume was automatically segmented using three-dimensional T1-weighted sequences. Physical function was assessed using the Short Physical Performance Battery (SPPB). Data were analyzed using general linear regression and mediation models.

**Results:**

Controlling for demographic characteristics, cardiovascular risk factors, stroke, disproportionately enlarged subarachnoid-space hydrocephalus (DESH), and total intracranial volume, per 1-ml increase in ChP volume was associated with β-coefficient of -0.24 (95% confidence interval: -0.37 to -0.11) for SPPB summary score, with the association being stronger in females (-0.40; -0.60 to -0.20) than in males (-0.17; -0.33 to -0.01) (p for ChP volume×sex interaction = 0.028). The associations were similar across three domains of balance, chair stand, and walking speed. In addition, enlarged ChP volume was associated with increased ventricular volume and white matter hyperintensity (WMH) volume. Mediation analysis suggested that lateral ventricular volume and periventricular WMH volume significantly mediated the association of ChP volume with the SPPB summary score, with the proportion of mediation being 54.22% and 14.48%, respectively.

**Conclusion:**

Larger ChP volume is associated with poorer physical function in older adults, especially in women. The association is largely mediated by lateral ventricular and periventricular WMH volumes.

**Graphical Abstract:**

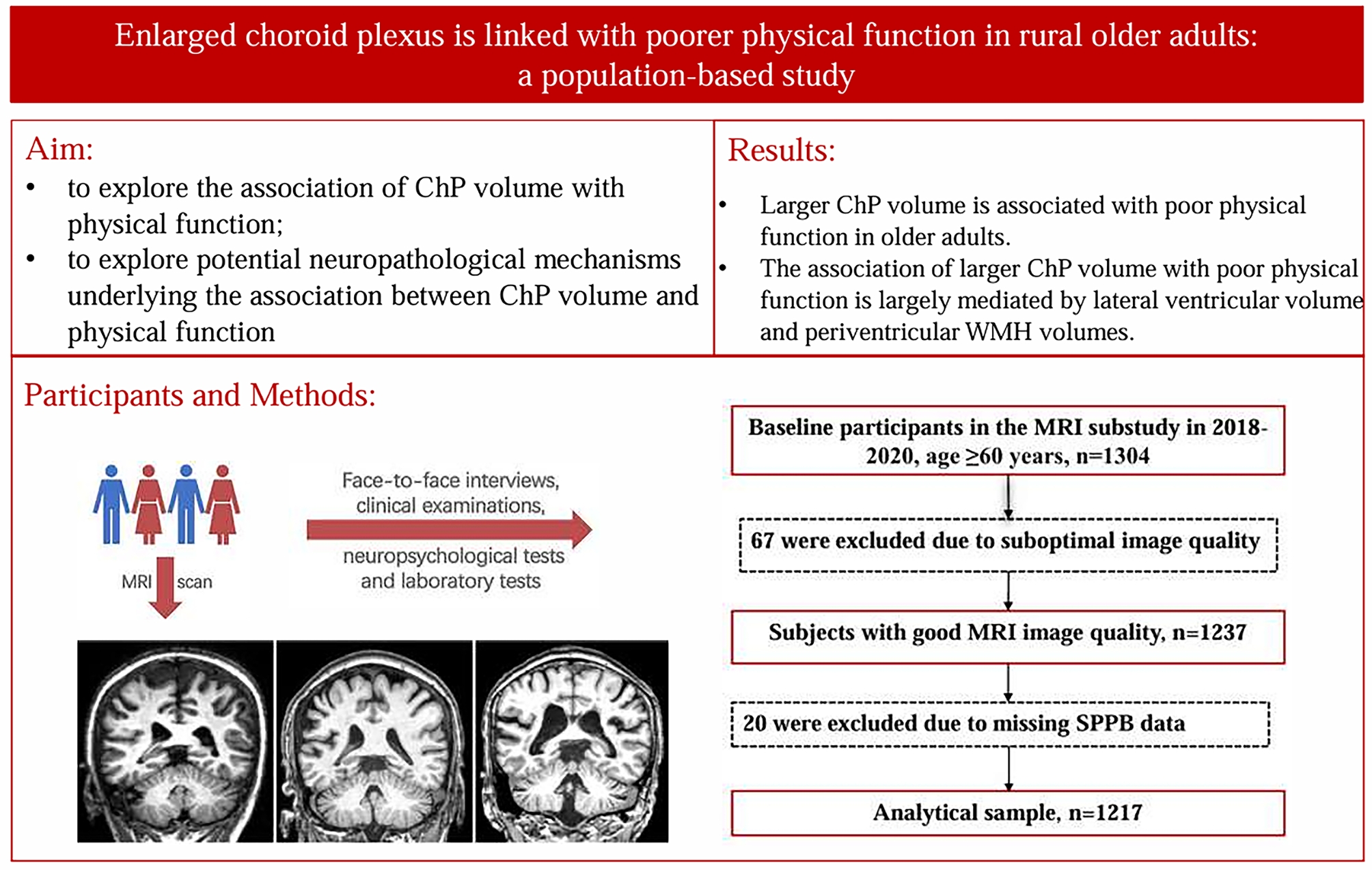

**Supplementary Information:**

The online version contains supplementary material available at 10.1186/s12987-025-00642-z.

## Background

The choroid plexus (ChP) is an endothelial-epithelial structure located in the lateral, third, and fourth ventricles that plays an important role in regulating brain homeostasis [1, 2]. The ChP is a multifunctional structure that not only produces cerebrospinal fluid (CSF) but also forms the blood-CSF barrier via tight junctions between choroidal epithelial cells. In addition, the ChP provides nutrients, a clearance system, and immune surveillance [1, 3]. In recent years, growing evidence has suggested a potential pathogenetic role of ChP and CSF formation abnormalities in neurodegenerative disorders [4–7]. Indeed, clinical-based neuroimaging studies have linked increased ChP volume with advanced stages of Alzheimer’s disease (AD) [8]. A population-based cross-sectional study of dementia-free older adults in Japan found that enlarged ChP volume was associated with poor cognitive function independent of brain parenchymal and CSF volumes [9]. In addition, a cross-sectional study of healthy adults suggested that enlarged ChP correlated with decreased ChP perfusion, increased ChP blood flow, and increased absolute CSF flow [10]. Thus, enlarged ChP may act as a compensatory mechanism to maintain sufficient CSF flow. However, chronic accumulation and malabsorption of CSF might contribute to the enlargement of ventricles. Previous studies have reported the association between ventricular dilatation and low physical function among community-dwelling older adults [11, 12]. Notably, low physical function assessed with the Short Physical Performance Battery (SPPB) is a well-established predictor for all-cause mortality [13]. In addition, several studies showed that ChP enlargement was associated with increased white matter hyperintensity (WMH) volume [14, 15]. Moreover, data from the Mayo Clinic Study of Aging (age ≥ 50 years) supported the association of greater WMH with physical dysfunction (e.g., impaired gait speed) [16]. However, the association of ChP volume with physical function and the underlying mechanisms of this association have not yet been investigated, especially in the general older population settings. This is important because understanding the possible mechanisms underlying physical impairment may help develop effective interventions to maintain physical function into older age. In addition, there are substantial sex differences in ChP volume and we previously reported that females had a lower SPPB score than males [17]. Thus, it is important to explore possible sex variations in the association of ChP volume with physical performance.

Therefore, in this population-based study, we sought to explore the association of ChP volume with physical function in rural-dwelling older adults while taking into account sex variations, and further to explore potential neuropathological mechanisms underlying the association between ChP volume and physical function.

## Methods

### Study design and participants

**Fig. 1:**
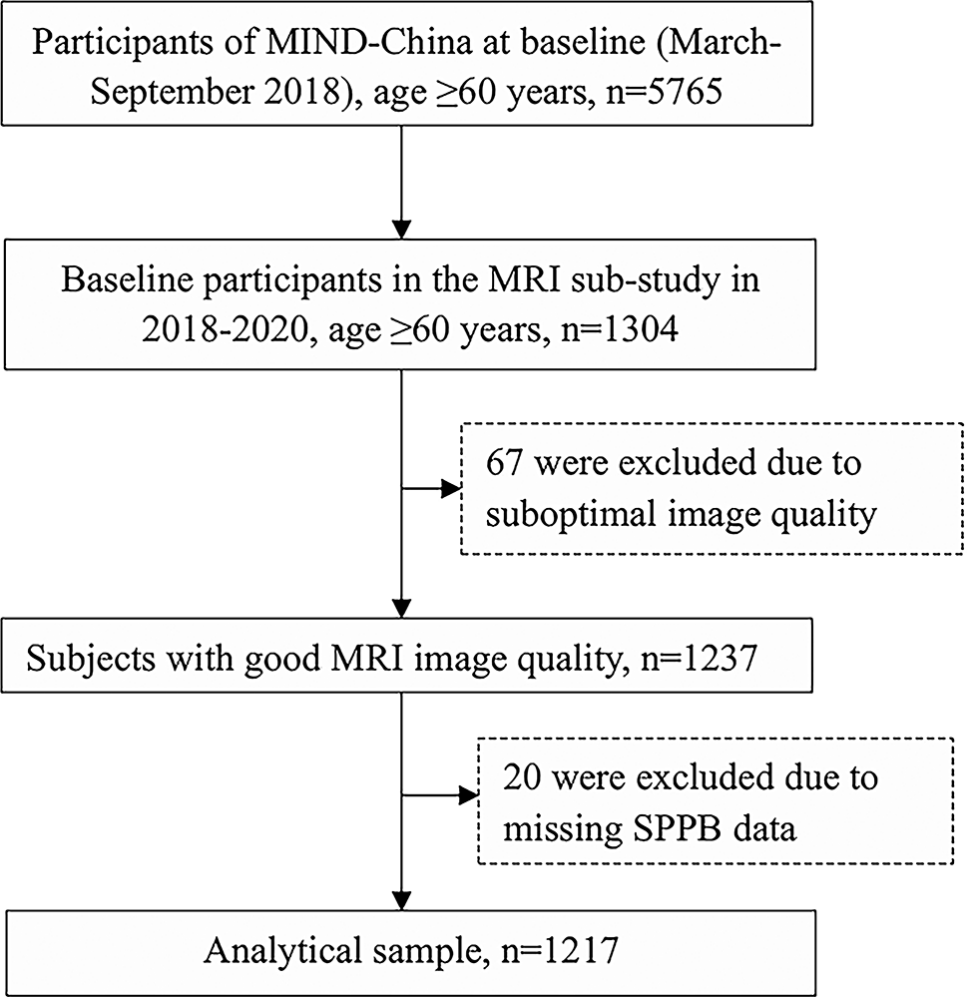
Flowchart of the study participants. MIND-China, Multimodal Intervention to delay Dementia and disability in rural China; MRI, magnetic resonance imaging; SPPB, Short Physical Performance Battery

This population-based cross-sectional study used data derived from the baseline assessments of the Multimodal Interventions to delay Dementia and disability in the rural China (MIND-China), a participating project in the World-Wide FINGERS Network, a global network for dementia risk reduction and prevention [18]. The MIND-China study targeted people who were aged ≥ 60 years by the end of 2017 and living in rural communities (52 villages) in western Shandong Province. In March-September 2018, 5765 participants (74.9% of all eligible persons) underwent the baseline examination in MIND-China [19]. Participants in the MRI sub-study were recruited from 26 villages that were randomly selected from all the 52 villages in a local town. In total, 1304 participants signed the informed consent and completed the structural brain MRI scans at either Southwestern Lu Hospital (n = 1178) or Liaocheng People’ Hospital (n = 126). Of these, 87 persons were excluded due to suboptimal image quality (n = 67) or missing data on physical function (n = 20), leaving 1217 participants for the analysis involving ChP volume and physical function (see Fig. 1 for flowchart of the study participants).

### Data collection and assessments

In March-September 2018, the trained medical staff collected data through face-to-face interviews, clinical and neurological examinations, and laboratory tests following a structured questionnaire, as previously reported [20, 21]. In brief, we collected data on demographic characteristics (e.g., age, sex, and education), lifestyle factors (e.g., smoking, alcohol intake, and physical exercise), and clinical factors (e.g., hypertension, diabetes, and hyperlipidemia). Peripheral blood samples were taken and then DNA was extracted. The *APOE* genotype was determined using the TaqMan single-nucleotide polymorphism (SNP) method [19] and was categorized as *ε*2/*ε*2 or *ε*2/*ε*3, *ε*3/*ε*3 (reference), and any *ε*4 allele. Education was categorized as no formal schooling, primary school, and middle school or above. Occupation was divided into farmers or non-farmers. Physical exercise was classified into engaging at least weekly versus less than weekly physical activity. Body mass index (BMI) was calculated as weight in kilogram divided by height in meters squared (kg/m^2^). Smoking and alcohol intake habits were dichotomized as current versus noncurrent smoking or alcohol intake. Hypertension was defined as self-reported physician diagnosis of hypertension or having blood pressure ≥ 140/90 mm Hg or current use of antihypertensive drugs. Diabetes mellitus was defined as self-reported history of diabetes or fasting blood glucose level ≥ 7.0 mmol/L or current use of glucose-lowering drugs or insulin injection. Hyperlipidemia was defined according to the 2016 Chinese guidelines for adults [22]: total cholesterol ≥ 6.2 mmol/L, triglycerides ≥ 2.3 mmol/L, LDL-C ≥ 4.1mmol/L, HDL-C ≤ 1.0 mmol/L or use of hypolipidemic agents. History of clinical stroke and Parkinson’s disease was ascertained according to self-reported medical history and neurological examination by a neurologist [23].

### Physical function assessment

The Short Physical Performance Battery (SPPB), which consists of a standing balancing test, a 4-meter walking test, and a 5 times chair sit-to-stand test, was used to evaluate physical function [21, 22]. Each of these three physical performance tests was given a score ranging from 0 to 4. The SPPB summary score was generated by summing up scores of the three subtests (range of the summary score: 0–12), with a higher score indicating better physical performance.

### Magnetic resonance imaging acquisition and processing

Structural brain images were acquired using the Philips Ingenia 3.0T MRI scanner (Philips Healthcare, Best, The Netherlands) in Southwestern Lu Hospital or the Philips Achieva 3.0T MRI scanner (Philips Healthcare, Best, the Netherlands) in Liaocheng People’ Hospital. The parameters of core MRI sequences were previously reported [24]. The 3D sT1-weighted images were processed using AccuBrain^®^ (BrainNow Medical Technology Ltd., Shenzhen, Guangdong, China) to obtain volumes of total intracranial volumes (ICV) and lateral ventricles. The segmentation of ChP in the lateral ventricles was performed using AccuBrain^®^ with a deep learning-based approach, as previously described [25]. Briefly, the ChP was manually labeled on 100 T1-weighted images by a trained neurologist (S.L.) and verified by a radiologist (G.T.) using ITK-SNAP software, version 4.0.1 (the Penn Image Computing and Science Laboratory, PICSL, USA) (http://www.itksnap.org*).* Then, the neural network was trained using these T1-weighted images, and the manually labeled ChP served as the ground truth. The network learns to recognize the unique features and patterns of ChP, allowing it to accurately segment this structure in new T1-weighted MRI scans. All automatic segmentation results were checked by two trained neurologists (S.L. and L.C.). The testing accuracy of an independent dataset (20 randomly selected subjects) achieved an average Dice coefficient of 0.82 and a volume correlation of 0.93 when comparing automatic labels with manual labels. The ChP volume was calculated automatically by scanning the automatic or manual label with the ITK-SNAP software.

WMH was defined as a hyperintense signal on T2 fluid-attenuated inversion recovery (FLAIR) images and WMH volume was acquired using AccuBrain, as previously reported [24]. In brief, T2 FLAIR images were used to calculate the signal contrast between normal brain tissue and WMH and then establish a signal threshold for WMH recognition, based on which WMHs were identified and extracted. Finally, the transformed T1-weighted brain structure mask extracted from our study samples was used to refine and localize WMHs. AccuBrain further automatically classified WMH into periventricular WMH and deep WMH following the rule of “continuity to ventricle,” while WMH < 10 mm distance from the ventricles was considered as periventricular WMH, otherwise as deep WMH [26].

Disproportionately enlarged subarachnoid-space hydrocephalus (DESH) is a neuroimaging phenotype of idiopathic normal-pressure hydrocephalus, originating from impaired CSF dynamics. We assessed the parameters of DESH related regions (i.e., the ventricular system, Sylvian fissure [SF], and subarachnoid space at high convexity and midline [SHM]) on T1-weighted images following a visual rating scale, as previously described [27]. In brief, DESH-related regions include the following three, (1) The Evans index (EI) value was the ratio of the maximum diameter of the frontal horns of the lateral ventricles to the maximum inner diameter of the skull on the transverse section. The ventricular system was classified as “not dilated” if EI ≤ 0.3 or “dilated” if EI > 0.3. Ventriculomegaly was defined as an EI > 0.3. (2) The SF was assessed in transverse and coronal sections. SF enlargement was rated as, 0 normal; 1, mildly dilated; 2, moderately dilated; and 3, severely dilated. Scores of 2 or 3 indicated enlarged SF. (3) The SHM was evaluated using transverse and coronal section images, and their tightness was rated as follows: 0, not tight; 1, moderately tight; and 2, severely tight. Scores of 1 or 2 constituted tight SHM. Participants meeting all criteria for ventriculomegaly, enlarged SF, and tight SHM were diagnosed with DESH.

### Statistical analysis

We performed descriptive statistics to present characteristics of study participants by sex-specific quartiles of ChP volume, in which we reported mean (standard deviation, SD) for continuous variables and frequencies (%) for categorical variables. We used one-way analysis of variance to compare continuous variables and the chi-square test to compare categorical variables. General linear regression analyses were performed to estimate β-coefficient and 95% confidence interval (CI) of SPPB score associated with ChP volume. The ChP volume was analyzed as both a continuous variable and a categorical variable of the sex-specific quartiles. We reported the main results from 2 different models, in which different potential confounding factors were controlled for step by step: model 1 was adjusted for age, sex, education, and ICV; model 2 was additionally adjusted for physical activity, BMI, smoking, alcohol intake, hypertension, diabetes, dyslipidemia, stroke, and disproportionately enlarged subarachnoid-space hydrocephalus, and *APOE* genotype. We tested statistical interaction of ChP volume with sex on physical performance. When a statistical interaction was detected, further stratifying analysis was performed to assess the direction and magnitude of the interaction. Finally, we performed the mediation analysis to examine the mediation effect of lateral ventricular volume and WMH volume on the association between ChP volume and physical function, with the bootstrapping of 5000 times, while controlling for age, sex, education, ICV, physical activity, BMI, smoking, alcohol intake, hypertension, diabetes, dyslipidemia, stroke, disproportionately enlarged subarachnoid-space hydrocephalus, and *APOE* genotype. Several sensitivity analyses were conducted to assess the robustness of the results. IBM SPSS Statistics 25.0 for Windows (IBM Corp., Armonk, NY, USA) and R version 4.1.3 (R Foundation for Statistical Computing, Vienna, Austria. https://www.R-project.org/) were used for all analyses and the package bruceR in R (Bao H, 2024; version 2024.6) https://cran.r-project.org/web/packages/bruceR/index.html was used for mediation analysis.

## Results

### Characteristics of study participants

The mean age of the 1217 participants was 69.46 (SD = 5.5; range, 60–86) years and 57.35% were women. The mean SPPB summary score was 10.11 (SD = 2.13; range, 0–12). Compared to participants with smaller ChP volume, those with larger ChP volume were older and had larger lateral ventricular volume and WMH volume (*P* < 0.001). There was a trend towards an increased ChP volume being significantly associated with a decrease in SPPB summary score and in the scores of the balance, chair stand, and walk tests (all *P* < 0.001) (Table 1).

**Table 1:** Characteristics of study participants by sex-specific quartiles of choroid plexus volume

	Total	Choroid plexus volume (ml), sex-specific quartiles^†^				
Characteristics	sample *n* = 1217	Q1, *n* = 305	Q2, *n* = 304	Q3, *n* = 303	Q4, *n* = 305	*P* value
Age (years)	69.46 (4.29)	68.36 (4.52)	68.86 (4.04)	69.64 (3.97)	71.00 (4.13)	< 0.001
Education (years)	3.56 (3.57)	3.54 (3.62)	3.51 (3.53)	3.51 (3.61)	3.68 (3.53)	0.922
Education, n (%)						0.265
Illiterate	422 (34.68)	115 (37.70)	106 (34.87)	105 (34.65)	96 (31.48)	
Primary school	550 (45.19)	127 (41.64)	131 (43.09)	135 (44.55)	157 (51.48)	
Middle school or above	245 (20.13)	63 (20.66)	67 (22.04)	63 (20.79)	52 (17.05)	
Occupation, n (%)						0.922
Farmer	984 (80.90)	245 (80.30)	243 (79.90)	243 (80.20)	253 (83.00)	
Non-farmer	230 (18.90)	59 (19.34)	60 (19.74)	59 (19.47)	52 (17.05)	
Physical exercise, n (%)						0.792
Weekly	240 (19.72)	55 (18.03)	60 (19.74)	60 (19.80)	65 (21.30)	
Less than weekly	977 (80.28)	250 (81.97)	244 (80.26)	243 (80.20)	240 (78.69)	
BMI (kg/m^2^)	25.00 (3.49)	24.57 (3.51)	25.01 (3.61)	25.06 (3.56)	25.36 (3.25)	0.048
Ever smoking, n (%)	440 (36.15)	111 (36.39)	104 (34.21)	116 (38.28)	109 (35.74)	0.771
Ever alcohol intake, n (%)	408 (33.53)	102 (33.44)	103 (33.88)	104 (34.32)	99 (32.46)	0.977
Hypertension, n (%)	813 (66.80)	193 (63.28)	195 (64.14)	208 (68.65)	217 (71.15)	0.163
Diabetes, n (%)	186 (15.28)	37 (12.13)	46 (15.13)	53 (17.49)	50 (16.39)	0.286
Hyperlipidemia, n (%)	296 (24.32)	66 (21.64)	69 (22.70)	80 (26.40)	81 (26.56)	0.366
Stroke	150 (14.03)	31 (10.16)	32 (10.53)	35 (11.55)	52 (17.05)	< 0.001
*APOE* ε4 allele, n (%)	182 (14.95)	40 (13.11)	38 (12.50)	51 (16.83)	53 (17.38)	0.405
DESH	23 (1.89)	3 (0.98)	5 (1.64)	4 (1.32)	11 (3.61)	0.078
WMH volume						
Total WMH volume	8.74 (11.02)	6.10 (8.92)	8.03 (10.93)	9.35 (10.50)	11.46 (12.75)	< 0.001
PWMH volume	7.42 (10.38)	5.00 (8.37)	6.67 (10.28)	7.97 (9.78)	10.00 (12.10)	< 0.001
DWMH volume	1.33 (1.41)	1.10 (1.25)	1.36 (1.41)	1.38 (1.47)	1.46 (1.48)	0.012
Ventricular volume						
Lateral ventricular volume	25.00 (11.60)	19.00 (8.43)	21.80 (9.06)	25.60 (9.90)	33.40 (13.04)	< 0.001
Inferior lateral ventricles volume	2.81 (0.93)	2.45 (0.68)	2.60 (0.74)	2.80 (0.78)	3.41 (1.13)	< 0.001
SPPB score						
Balance score	3.63 (0.82)	3.76 (0.60)	3.62 (0.88)	3.68 (0.80)	3.48 (0.93)	< 0.001
Chair stand score	2.88 (1.19)	3.03 (1.12)	2.93 (1.16)	2.87 (1.19)	2.70 (1.25)	0.007
Walk test score	3.57 (0.72)	3.64 (0.65)	3.63 (0.67)	3.56 (0.80)	3.47 (0.76)	0.007
Summary score	10.11 (2.13)	10.51 (1.74)	10.17 (2.12)	10.12 (2.30)	9.67 (2.26)	< 0.001

Abbreviations: BMI, body mass index; *APOE*, apolipoprotein E gene; DESH, disproportionately enlarged subarachnoid-space hydrocephalus; WMH, white matter hyperintensity; PWMH, periventricular white matter hyperintensity; DWMH, deep white matter hyperintensity; SPPB, Short Physical Performance Battery

Note: Data were mean (standard deviation), unless otherwise specified. The number of participants with missing values was 3 for occupation, 6 for body mass index, 13 for alcohol drinking, 13 for hypertension, 41 for *APOE* genotype, 148 for stroke, 48 for balance score, 1 for chair stand score, 5 for walk test score and 47 for SPPB summary score. In the subsequent analyses, categorical variable with missing values was replaced with a dummy variable and continuous variable with missing values was replaced with a mean value

^†^The cut-offs of quartiles for total choroid plexus volume in females were < 2.68 ml (Q1), 2.68–3.30 ml (Q2), 3.30–3.87 ml (Q3), and > 3.87 ml (Q4); the corresponding cut-offs in males were < 3.72 ml (Q1), 3.72–4.49 ml (Q2), 4.49–5.10 ml (Q3), and > 5.10 ml (Q4)

### Associations of ChP volume with physical function

Controlling for age, sex, education, and ICV, a greater ChP volume was significantly associated with lower SPPB summary score (*P* < 0.05) (Table 2, model 1). Adjusting for additional potential confounding factors, the association remained statistically significant (Table 2, model 2). When ChP volume was analyzed as a categorical variable (sex-specific quartiles), compared to the lowest quartile, the top quartile of ChP volume was significantly associated with a reduced SPPB summary score (*P* < 0.01) (Table 2, model 1); adjusting for additional potential confounding factors, the highest quartile of ChP volume remained significantly associated with the lower SPPB summary score (*P* < 0.01) (Table 2, model 2).

**Table 2:** Associations of choroid plexus volume with SPPB summary score (n = 1170)

Choroid plexus volume as an exposure variable	β coefficient (95% confidence interval), SPPB summary score	
	Model 1	Model 2
ChP volume, continuous	-0.29 (-0.42, -0.16)^**^	-0.24 (-0.37, -0.11)^**^
ChP volume, sex-specific quartiles^†^		
Q1 (*n* = 288)	0.00 (reference)	0.00 (reference)
Q2 (*n* = 295)	-0.32 (-0.65, 0.01)	-0.28 (-0.60, 0.05)
Q3 (*n* = 288)	-0.29 (-0.63, 0.05)	-0.27 (-0.61, 0.07)
Q4 (*n* = 299)	-0.65 (-1.01, -0.30)^**^	-0.52 (-0.87, -0.17)^**^
P for linear trend	< 0.001	0.007

Abbreviations: ChP, choroid plexus volume; SPPB, Short Physical Performance Battery

Note: Data were β coefficients (95% confidence intervals) of SPPB summary score associated with choroid plexus volume as a continuous variable (per 1-ml increase) and sex-specific quartiles derived from two different models. Model 1 was controlled for age, sex, education, and total intracranial volume; model 2 was additionally adjusted for smoking, alcohol intake, body mass index, physical exercise, hypertension, diabetes, dyslipidemia, *APOE* genotype, stroke, and disproportionately enlarged subarachnoid-space hydrocephalus

^†^The cut-offs of quartiles for total choroid plexus volume in females were < 2.68 ml (Q1), 2.68–3.30 ml (Q2), 3.30–3.87 ml (Q3), and > 3.87 ml (Q4) and the corresponding cut-offs in males were < 3.72 ml (Q1), 3.72–4.49 ml (Q2), 4.49–5.10 ml (Q3), and > 5.10 ml (Q4)

^*^P < 0.05, ^**^P < 0.01

**Fig. 2:**
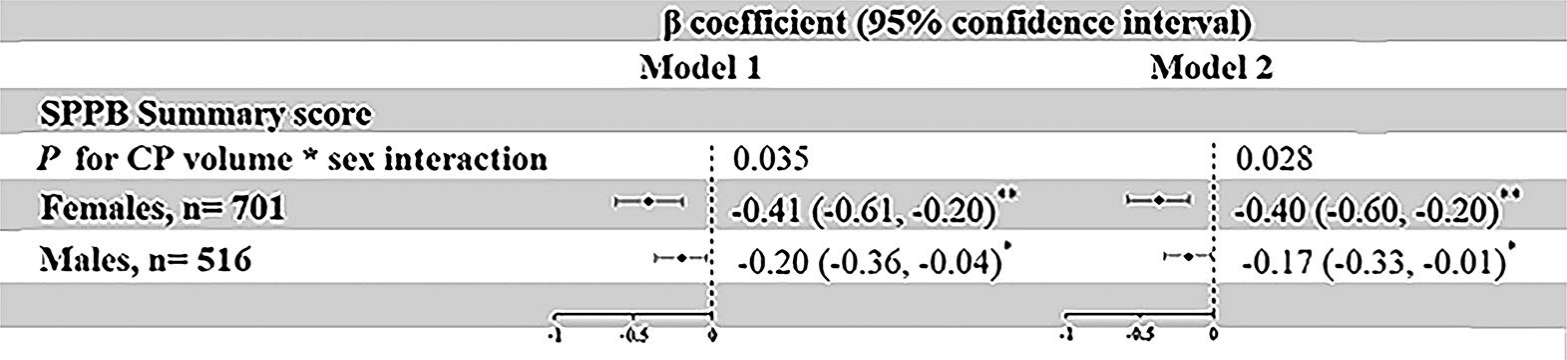
Associations of choroid plexus volume with physical function assessed with SPPB summary score by sex. Abbreviations: ChP, choroid plexus; SPPB, Short Physical Performance Battery. Note: Model 1 was controlled for age, education, and total intracranial volume; model 2 was additionally adjusted for smoking, alcohol intake, body mass index, physical exercise, hypertension, diabetes, dyslipidemia, *APOE* genotype, stroke, and disproportionately enlarged subarachnoid-space hydrocephalus. ^*^P < 0.05, ^**^P < 0.01

We detected statistical interactions of ChP volume with sex on the SPPB summary score (*P*for ChP volume×sex interactions < 0.05). Stratified analysis by sex suggested that the association of larger ChP volume with lower SPPB summary score was stronger in females than in males in the multivariable-adjusted model (Fig. 2, model 1); the associations remained significant in both females and males after additionally adjusting for additional potential confounding factors (Fig. 2, model 2).

### Associations of choroid plexus volume with WMH volume and ventricular volume

Controlling for age, sex, education, and ICV, a greater ChP volume was significantly associated with larger total WMH volume and periventricular WMH volume (both *P* < 0.01) (Table 3, model 1). Adjusting for additional potential confounding factors, the association remained statistically significant (Table 3, model 2). However, larger ChP volume was not significantly associated with deep WMH volume (*P* > 0.05) (Table 3, models 1 and 2). In addition, after adjusting for age, sex, education, and ICV, larger ChP volume was also significantly related to larger lateral ventricular volume and inferior lateral ventricular volume (both *P* < 0.01) (Table 3, model 1). Adjusting for additional potential confounding factors, the associations remained statistically significant (Table 3, model 2).

**Table 3:** Associations of choroid plexus volume with white matter hyperintensity volume and ventricular volume (*n* = 1165)

MRI markers	β coefficient (95% CI), MRI markers	
	Model 1	Model 2
**WMH volume** (ml)		
Total WMH volume	1.48 (0.81, 2.16) ^**^	1.23 (0.60, 1.95) ^**^
Periventricular WMH volume	1.41 (0.78, 2.05) ^**^	1.21 (0.58, 1.84) ^**^
Deep WMH volume	0.07 (-0.02, 0.16)	0.07 (-0.02, 0.16)
**Ventricular volume** (ml)		
Lateral ventricular volume	4.94 (4.37, 5.52) ^**^	4.67 (4.11, 5.23) ^**^
Inferior lateral ventricles volume	0.34 (0.30, 0.39) ^**^	0.33 (0.28, 0.37) ^**^

Abbreviations: MRI, magnetic resonance imaging; WMH, white matter hyperintensity; CI, Confidence Interval; SPPB, Short Physical Performance Battery

Note: Data were β coefficients (95% confidence intervals) of volumetric measures of MRI markers associated with per 1-ml increase in choroid plexus volume derived from two different models. Model 1 was controlled for age, sex, education, and total intracranial volume; model 2 was additionally adjusted for smoking, alcohol intake, body mass index, physical exercise, hypertension, diabetes, dyslipidemia, *APOE* genotype, stroke, and disproportionately enlarged subarachnoid-space hydrocephalus

^*^P < 0.05, ^**^P < 0.01

### Mediation of lateral ventricular volume and periventricular WMH volume in the association of ChP volume with physical function

**Fig. 3:**
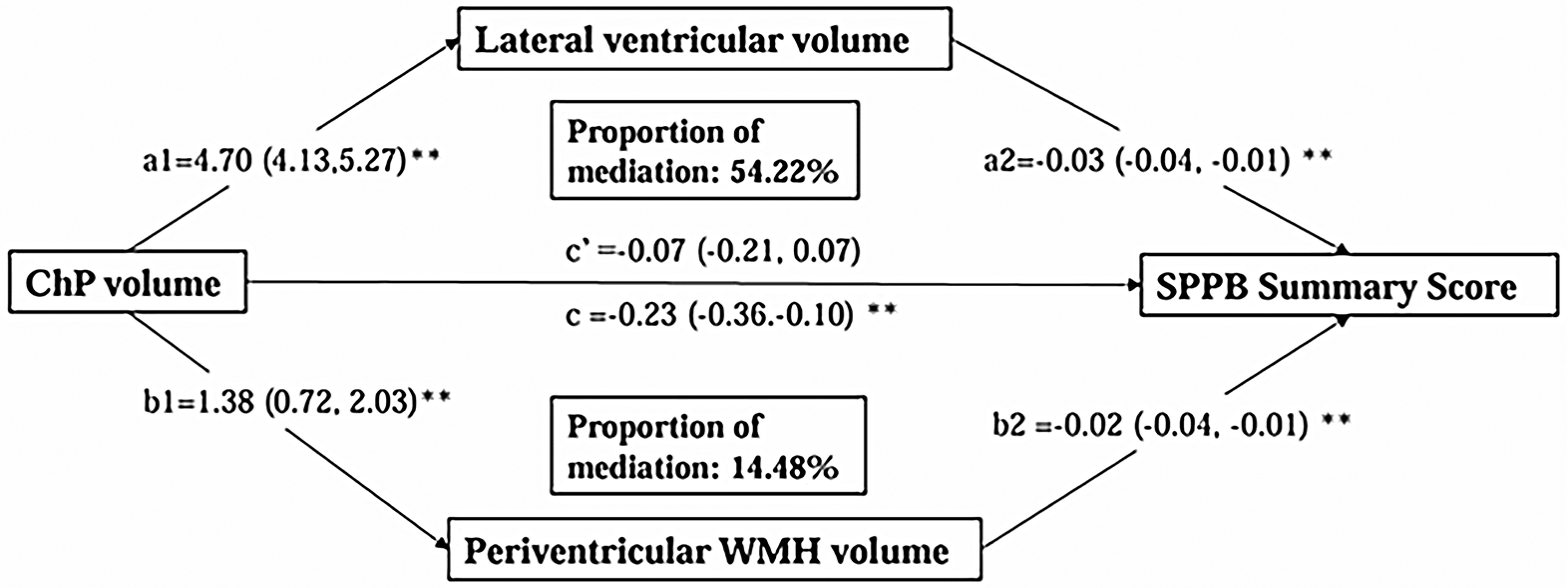
Mediation effects of MRI markers on the associations of choroid plexus volume with physical function. Abbreviations: ChP, choroid plexus; SPPB, Short Physical Performance Battery.Note: The model was adjusted for age, sex, education, total intracranial volume, body mass index, physical exercise, ever smoking, alcohol intake, hypertension, diabetes, dyslipidemia, APOE genotype, stroke, and disproportionately enlarged subarachnoid-space hydrocephalus. ^*^P < 0.05, ^**^P < 0.01

We performed parallel mediation analysis to further explore the extent to which ventricular volume and WMH volume could mediate the cross-sectional association between ChP volume and physical function. Controlling for demographics, vascular risk factors, total ICV, physical exercise, *APOE* genotype, stroke, and disproportionately enlarged subarachnoid-space hydrocephalus, larger lateral ventricular volume and periventricular WMH volume, which were significantly associated with lower SPPB summary score (both *P* < 0.01), could significantly mediate 54.22% and 14.48%, respectively, of the association between ChP volume and the SPPB summary score (Fig. 3).

### Sensitivity analysis

The following sensitivity analyses were performed to further assess the robust association between ChP volume and physical function. First, we examined the relationship of ChP volume with scores of three SPPB subtests (e.g., the balance, chair stand, and walking speed tests). Controlling for potential confounding factors, a greater ChP volume was significantly associated with lower scores of three SPPB subtests (all *P* < 0.05) (Table S1, Models 1 and 2). When ChP volume was analyzed as a categorical variable (sex-specific quartiles), compared to the lowest quartile, the top quartile of ChP volume was significantly associated with a lower balance score and a reduced chair stand test score (all *P* < 0.01) (Table S1, Models 1 and 2). Furthermore, we repeated the analysis by excluding participants with dementia (n = 29) and Parkinson’s Disease (n = 2). The relationships of ChP volume, both as continuous and categorical variables, with SPPB summary score remained statistically significant after adjusting for potential confounding factors (Table S2, Models 1 and 2). We also repeated the analysis by further excluding 272 participants with mild cognitive impairment from the dementia-free participants. The associations of ChP volume, as a continuous variable, with SPPB summary score remained statistically significant (*P* = 0.007, data not shown). Finally, we repeated the analysis by excluding participants from Liaocheng People’ Hospital (n = 97). The relationships of ChP volume, both as continuous and categorical variables, with SPPB summary score were similar to results from the main analysis (Table S3, Models 1 and 2).

## Discussion

The main findings from this population-based study of rural-dwelling older adults in China can be summarized as follows: (1) the larger ChP volume was associated with lower physical function; (2) the associations of larger ChP volume with worse physical function varied with sex, such that the associations were evident mainly in females; and (3) the association of enlarged ChP volume with poor physical function was largely mediated by the lateral ventricular volume and periventricular WMH.

Previously, a clinical-based study of patients with early-stage Parkinson’s disease showed that larger ChP volume was associated with motor deficits [28]. In a population-based study of dementia-free older adults in Japan, an increased ChP volume was found to be cross-sectionally correlated with a lower Mini-Mental State Examination score [9]. To the best of our knowledge, our study is the first population-based study that explores the association of ChP volume with physical function among older adults. We found that a greater ChP volume was associated with poorer physical function, including impaired balance, chair stand, and gait speed. We further explored the potential mechanisms linking enlarged ChP volume with physical dysfunction. Firstly, we explored whether the association of enlarged ChP volume with poor physical function was largely dependent on ventricular volume. This is important because pathophysiologically, enlarged ChP and lateral ventricles are closely related [10, 29], and several population-based studies have reported the association between ventricular volume and physical function in older adults. For instance, the cross-sectional data from the US Cardiovascular Health Study showed that larger ventricular volume was associated with balance disorders [30]. Furthermore, the cross-sectional data from the Icelandic Age, Gene/Environment Susceptibility-Reykjavik Study suggested that ventricular dilation was associated with gait impairment [31]. Similarly, the Mayo Clinic Study of Aging suggested that ventricular enlargement was strongly associated with slower gait speed [12]. Therefore, it is plausible to hypothesize that the association of enlarged ChP with physical impairment could be, at least partly, attributable to enlarged lateral ventricle. Then, we explored the role of WMH volume in the association of enlarged ChP volume with poor physical function. Previously, population-based studies have shown that enlarged ChP volume are closely related to larger WMH volume [14, 15]. Data from the Alzheimer’s Disease Neuroimaging Initiative also showed that a greater WMH burden was associated with enlarged ChP volume in older adults [32]. Moreover, several studies have linked increased WMH volume with physical dysfunction in older adults [33–37]. For example, the cross-sectional data from the Guangdong SVD study in China showed that WMH is associated with motor dysfunction [34]. In support of this hypothesis, our population-based study indeed revealed for the first time that lateral ventricular volume and periventricular WMH could partly mediate the cross-sectional association between enlarged ChP volume and low physical function. The mechanisms underlying the ChP volume-physical function association among older adults are not fully understood. Previous studies have shown that neuroinflammation is involved in abnormal deposition of lipofuscin and amyloid in ChP epithelial cells, which in turn could result in epithelial edema and ChP enlargement [38, 39]. In addition, pro-inflammatory cytokines might promote the differentiation or proliferation of nonimmune cells, and further lead to ChP enlargement [40]. Enlarged ventricles may impede frontal projections and midbrain, the vital anatomical structures for regulating balance and gait, thus leading to physical dysfunction (e.g., impaired balance and gait speed) [41–43]. Moreover, neuroinflammation is known to play a crucial role in the pathogenesis of cerebral small vessel disease (CSVD) [44]. Gait impairment associated with CSVD is likely caused by interrupted frontal cortical–subcortical circuits and decreased connectivity of cerebral white matter tracts in older adults [45].

We detected the sex-varying association between larger ChP volume and lower physical performance, such that a larger ChP volume showed a stronger association with lower physical performance in females than in males. The biological mechanisms underlying the sex-varying association between the ChP volume and physical function are unknown. In the MIND-China cohort, the prevalence of obesity, hypertension, diabetes, and dyslipidemia were higher in females than males [19, 46, 47]. Previous studies have correlated cardiometabolic risk factors (e.g., hypertension and obesity) with enlarged ChP volume [8, 48]. However, our study indicated that the sex differences in the association of larger ChP volume with poorer physical function was present independent of major cardiometabolic risk factors. Further research may help elucidate neuropathological mechanisms underlying the sex differences in their association.

This large-scale population-based MRI study engaged older adults who were living in rural communities in China, a sociodemographic group that has been underrepresented in research of aging and physical function. However, our study also has limitations. First, a temporal relationship of ChP volume with poor physical performance cannot be determined owing to the nature of cross-sectional design. Second, we did not use the gold standard manual annotation for ChP segmentation due to time-consuming and cumbersome in a large sample, instead, we used the deep learning models to automatically segment ChP volume, which is considered a simple and highly reliable solution [25]. Third, the study sample was drawn from a single rural area of western Shandong Province where a considerable proportion of participants had received no or very limited school education. This should be kept in mind when generalizing the results of our study to other populations.

In conclusion, this population-based cross-sectional study provides evidence in favor of the hypothesis that enlarged ChP volume is associated with poorer physical function in rural older adults. Of note, we revealed that the lateral ventricular volume and WMH volume could largely mediate the ChP volume-physical function association, suggesting that ventricular enlargement due to ChP malfunction may be a biological mechanism or pathway underlying physical dysfunction. Future prospective follow-up studies are warranted to further explore the potential causal relationship of ChP volume with subsequent consequences of physical function as well as the mechanisms underlying their association in older adults.

## Electronic supplementary material

Below is the link to the electronic supplementary material.

Supplementary Material 1

## Data Availability

No datasets were generated or analysed during the current study.

## Abbreviations

ChP: Choroid plexus
CSF: Cerebrospinal fluid
SPPB: Short Physical Performance Battery
MIND-China: The Multimodal Interventions to delay Dementia and disability in rural China
AD: Alzheimer’s disease
MCI: Mild cognitive impairment
BMI: Body mass index
ICV: Intracranial volumes
DESH: Disproportionately enlarged subarachnoid-space hydrocephalus
CI: Confidence interval

## References

[1] Lun MP, Monuki ES, Lehtinen MK. Development and functions of the choroid plexus-cerebrospinal fluid system. Nat Rev Neurosci. 2015;16(8):445–57. 26174708 10.1038/nrn3921 PMC4629451

[2] Damkier HH, Brown PD, Praetorius J. Cerebrospinal fluid secretion by the choroid plexus. Physiol Rev. 2013;93(4):1847–92. 24137023 10.1152/physrev.00004.2013

[3] Saunders NR, Dziegielewska KM, Fame RM, Lehtinen MK, Liddelow SA. The choroid plexus: a missing link in our Understanding of brain development and function. Physiol Rev. 2023;103(1):919–56. 36173801 10.1152/physrev.00060.2021 PMC9678431

[4] Solár P, Zamani A, Kubíčková L, Dubový P, Joukal M. Choroid plexus and the blood-cerebrospinal fluid barrier in disease. Fluids Barriers CNS. 2020;17(1):35. 32375819 10.1186/s12987-020-00196-2 PMC7201396

[5] Municio C, Carrero L, Antequera D, Carro E. Choroid plexus aquaporins in CSF homeostasis and the glymphatic system: their relevance for Alzheimer’s disease. Int J Mol Sci. 2023;24(1):878. 10.3390/ijms24010878 PMC9821203 36614315

[6] Tadayon E, Pascual-Leone A, Press D, Santarnecchi E. Choroid plexus volume is associated with levels of CSF proteins: relevance for Alzheimer’s and Parkinson’s disease. Neurobiol Aging. 2020;89:108–17. 32107064 10.1016/j.neurobiolaging.2020.01.005 PMC9094632

[7] Čarna M, Onyango IG, Katina S, Holub D, Novotny JS, Nezvedova M, et al. Pathogenesis of Alzheimer’s disease: involvement of the choroid plexus. Alzheimer’s Dement. 2023;19(8):3537–54. 10.1002/alz.12970 PMC10634590 36825691

[8] Choi JD, Moon Y, Kim HJ, Yim Y, Lee S, Moon WJ. Choroid plexus volume and permeability at brain MRI within the alzheimer disease clinical spectrum. Radiology. 2022;304(3):635–45. 35579521 10.1148/radiol.212400

[9] Hidaka Y, Hashimoto M, Suehiro T, Fukuhara R, Ishikawa T, Tsunoda N, et al. Association between choroid plexus volume and cognitive function in community-dwelling older adults without dementia: a population-based cross-sectional analysis. Fluids Barriers CNS. 2024;21(1):101. 39696504 10.1186/s12987-024-00601-0 PMC11654186

[10] Eisma JJ, McKnight CD, Hett K, Elenberger J, Song AK, Stark AJ, et al. Choroid plexus perfusion and bulk cerebrospinal fluid flow across the adult lifespan. J Cereb Blood Flow Metab. 2023;43(2):269–80. 10.1177/0271678X221129101 PMC9903224 36200473

[11] Annweiler C, Montero-Odasso M, Bartha R, Drozd J, Hachinski V, Beauchet O. Association between gait variability and brain ventricle attributes: a brain mapping study. Exp Gerontol. 2014;57:256–63. 24971908 10.1016/j.exger.2014.06.015

[12] Crook JE, Gunter JL, Ball CT, Jones DT, Graff-Radford J, Knopman DS, et al. Linear vs volume measures of ventricle size: relation to present and future gait and cognition. Neurology. 2020;94(5):e549–56. 31748251 10.1212/WNL.0000000000008673 PMC7080290

[13] Pavasini R, Guralnik J, Brown JC, di Bari M, Cesari M, Landi F, et al. Short physical performance battery and all-cause mortality: systematic review and meta-analysis. BMC Med. 2016;14(1):215. 28003033 10.1186/s12916-016-0763-7 PMC5178082

[14] Li Y, Zhou Y, Zhong W, Zhu X, Chen Y, Zhang K, et al. Choroid plexus enlargement exacerbates white matter hyperintensity growth through glymphatic impairment. Ann Neurol. 2023;94(1):182–95. 36971336 10.1002/ana.26648

[15] Li C, Zhang H, Wang J, Han X, Liu C, Li Y, et al. Choroid plexus volume in rural Chinese older adults: distribution and association with cardiovascular risk factors and cerebral small vessel disease. J Am Heart Assoc. 2024;13(21):e035941. 39424375 10.1161/JAHA.124.035941 PMC11935722

[16] Raghavan S, Przybelski SA, Lesnick TG, Fought AJ, Reid RI, Gebre RK, et al. Vascular risk, gait, behavioral, and plasma indicators of VCID. Alzheimers Dement. 2024;20(2):1201–13. 37932910 10.1002/alz.13540 PMC10916988

[17] Han X, Wang X, Wang C, Wang P, Han X, Zhao M, et al. Accelerometer-assessed sedentary behaviour among Chinese rural older adults: patterns and associations with physical function. J Sports Sci. 2022;40(17):1940–9. 36112669 10.1080/02640414.2022.2122321

[18] Kivipelto M, Mangialasche F, Snyder HM, Allegri R, Andrieu S, Arai H, et al. World-wide fingers network: A global approach to risk reduction and prevention of dementia. Alzheimer’s Dement. 2020;16(7):1078–94. 10.1002/alz.12123 PMC9527644 32627328

[19] Cong L, Ren Y, Wang Y, Hou T, Dong Y, Han X, et al. Mild cognitive impairment among rural-dwelling older adults in China: A community-based study. Alzheimer’s Dement. 2023;19(1):56–66. 10.1002/alz.12629 PMC10078715 35262288

[20] Han XD, Li YJ, Wang P, Han XL, Zhao MQ, Wang JF, et al. Insulin resistance-varying associations of adiposity indices with cerebral perfusion in older adults: A Population-Based study. J Nutr Health Aging. 2023;27(3):219–27.36973931 10.1007/s12603-023-1894-2

[21] Song L, Han X, Li Y, Han X, Zhao M, Li C, et al. Thalamic gray matter volume mediates the association between KIBRA polymorphism and olfactory function among older adults: a population-based study. Cereb Cortex. 2023;33(7):3664–73. 35972417 10.1093/cercor/bhac299 PMC10068283

[22] Jun-Ren ZHU, et al. 2016 Chinese guidelines for the management of dyslipidemia in adults. J Geriatr Cardiol. 2018;15(1):1–29. 10.11909/j.issn.1671-5411.2018.01.011 PMC5803534 29434622

[23] Ren Y, Li Y, Tian N, Liu R, Dong Y, Hou T, et al. Multimorbidity, cognitive phenotypes, and Alzheimer’s disease plasma biomarkers in older adults: A population-based study. Alzheimers Dement. 2024;20(3):1550–61. 38041805 10.1002/alz.13519 PMC10984420

[24] Dong Y, Li Y, Liu K, Han X, Liu R, Ren Y, et al. Anosmia, mild cognitive impairment, and biomarkers of brain aging in older adults. Alzheimers Dement. 2023;19(2):589–601. 36341691 10.1002/alz.12777

[25] Yazdan-Panah A, Schmidt-Mengin M, Ricigliano VAG, Soulier T, Stankoff B, Colliot O. Automatic segmentation of the choroid plexuses: method and validation in controls and patients with multiple sclerosis. Neuroimage Clin. 2023;38:103368. 36913908 10.1016/j.nicl.2023.103368 PMC10011049

[26] Griffanti L, Jenkinson M, Suri S, Zsoldos E, Mahmood A, Filippini N, et al. Classification and characterization of periventricular and deep white matter hyperintensities on MRI: A study in older adults. NeuroImage. 2018;170:174–81. 28315460 10.1016/j.neuroimage.2017.03.024

[27] Hidaka Y, Hashimoto M, Suehiro T, Fukuhara R, Ishikawa T, Tsunoda N, et al. Impact of age on the cerebrospinal fluid spaces: high-convexity and medial subarachnoid spaces decrease with age. Fluids Barriers CNS. 2022;19(1):82. 36307853 10.1186/s12987-022-00381-5 PMC9615391

[28] Jeong SH, Park CJ, Jeong HJ, Sunwoo MK, Ahn SS, Lee SK, et al. Association of choroid plexus volume with motor symptoms and dopaminergic degeneration in Parkinson’s disease. J Neurol Neurosurg Psychiatry. 2023;94(12):1047–55. 10.1136/jnnp-2023-331170 37399288

[29] Yamada S, Ishikawa M, Nozaki K. Exploring mechanisms of ventricular enlargement in idiopathic normal pressure hydrocephalus: a role of cerebrospinal fluid dynamics and motile cilia. Fluids Barriers CNS. 2021;18(1):20. 33874972 10.1186/s12987-021-00243-6 PMC8056523

[30] Tell GS, Lefkowitz DS, Diehr P, Elster AD. Relationship between balance and abnormalities in cerebral magnetic resonance imaging in older adults. Arch Neurol. 1998;55(1):73–9. 9443713 10.1001/archneur.55.1.73

[31] Palm WM, Saczynski JS, van der Grond J, Sigurdsson S, Kjartansson O, Jonsson PV, et al. Ventricular dilation: association with gait and cognition. Ann Neurol. 2009;66(4):485–93. 19847895 10.1002/ana.21739 PMC4530517

[32] Hong H, Hong L, Luo X, Zeng Q, Li K, Wang S, et al. The relationship between amyloid pathology, cerebral small vessel disease, glymphatic dysfunction, and cognition: a study based on Alzheimer’s disease continuum participants. Alzheimers Res Ther. 2024;16(1):43. 38378607 10.1186/s13195-024-01407-w PMC10877805

[33] Hairu R, Close JCT, Lord SR, Delbaere K, Wen W, Jiang J, et al. The association between white matter hyperintensity volume and gait performance under single and dual task conditions in older people with dementia: A cross-sectional study. Arch Gerontol Geriatr. 2021;95:104427. 34015687 10.1016/j.archger.2021.104427

[34] Zhao X, Zuo M, Zhan F, Fan P, Liu S, Taylor M, et al. Cognition mediates the relationship between white matter hyperintensity and motor function in patients with cerebral small vessel disease: a cross-sectional study. Quant Imaging Med Surg. 2024;14(10):7306–17. 39429558 10.21037/qims-24-1058 PMC11485344

[35] Taylor ME, Lord SR, Delbaere K, Wen W, Jiang J, Brodaty H, et al. White matter hyperintensities are associated with falls in older people with dementia. Brain Imaging Behav. 2019;13(5):1265–72. 30145714 10.1007/s11682-018-9943-8

[36] Zheng JJ, Lord SR, Close JC, Sachdev PS, Wen W, Brodaty H, et al. Brain white matter hyperintensities, executive dysfunction, instability, and falls in older people: a prospective cohort study. J Gerontol Biol Sci Med Sci. 2012;67(10):1085–91. 10.1093/gerona/gls063 22403055

[37] Whitman GT, Tang Y, Lin A, Baloh RW. A prospective study of cerebral white matter abnormalities in older people with gait dysfunction. Neurology. 2001;57(6):990–4. 11571322 10.1212/wnl.57.6.990

[38] Brkic M, Balusu S, Van Wonterghem E, Gorlé N, Benilova I, Kremer A, et al. Amyloid Β oligomers disrupt Blood-CSF barrier integrity by activating matrix metalloproteinases. J Neurosci. 2015;35(37):12766–78. 10.1523/JNEUROSCI.0006-15.2015 PMC6795210 26377465

[39] Fleischer V, Gonzalez-Escamilla G, Ciolac D, Albrecht P, Küry P, Gruchot J, et al. Translational value of choroid plexus imaging for tracking neuroinflammation in mice and humans. Proc Natl Acad Sci U S A. 2021;118(36). 10.1073/pnas.2025000118 PMC8433504 34479997

[40] Tanaka T, Narazaki M, Kishimoto T. IL-6 in inflammation, immunity, and disease. Cold Spring Harb Perspect Biol. 2014;6(10):a016295. 25190079 10.1101/cshperspect.a016295 PMC4176007

[41] Bradley WG. Normal pressure hydrocephalus: new concepts on etiology and diagnosis. AJNR Am J Neuroradiol. 2000;21(9):1586–90. 11039335 PMC8174878

[42] Ishii M, Kawamata T, Akiguchi I, Yagi H, Watanabe Y, Watanabe T, et al. Parkinsonian symptomatology may correlate with ct findings before and after shunting in idiopathic normal pressure hydrocephalus. Parkinsons Dis. 2010;10:2010:201089. 10.4061/2010/201089 PMC2951141 20948890

[43] Lee PH, Yong SW, Ahn YH, Huh K. Correlation of midbrain diameter and gait disturbance in patients with idiopathic normal pressure hydrocephalus. J Neurol. 2005;252(8):958–63. 15834647 10.1007/s00415-005-0791-2

[44] Walsh J, Tozer DJ, Sari H, Hong YT, Drazyk A, Williams G, et al. Microglial activation and blood-brain barrier permeability in cerebral small vessel disease. Brain. 2021;144(5):1361–71. 34000009 10.1093/brain/awab003 PMC8874873

[45] Parihar R, Mahoney JR, Verghese J. Relationship of gait and cognition in the elderly. Curr Transl Geriatr Exp Gerontol Rep. 2013;2(3). 10.1007/s13670-013-0052-7 PMC3859437 24349877

[46] Han X, Jiang Z, Li Y, Wang Y, Liang Y, Dong Y, et al. Sex disparities in cardiovascular health metrics among rural-dwelling older adults in China: a population-based study. BMC Geriatr. 2021;21(1):158. 33663413 10.1186/s12877-021-02116-x PMC7934439

[47] Wang Y, Han X, Zhang X, Zhang Z, Cong L, Tang S, et al. Health status and risk profiles for brain aging of rural-dwelling older adults: data from the interdisciplinary baseline assessments in MIND-China. Alzheimer’s Dement (New York N Y). 2022;8(1):e12254. 10.1002/trc2.12254 PMC9009233 35441085

[48] Alisch JSR, Egan JM, Bouhrara M. Differences in the choroid plexus volume and microstructure are associated with body adiposity. Front Endocrinol. 2022;13:984929. 10.3389/fendo.2022.984929 PMC9606414 36313760

