# Supplementary material for: Enlarged choroid plexus is linked with poorer physical function in rural older adults: a population-based study
Source: Fluids Barriers CNS. 2025 Apr 3;22:33. doi: 10.1186/s12987-025-00642-z (PMC11966853; doi:10.1186/s12987-025-00642-z)
Supplement: Supplementary file 1 — Supplementary Material 1 [file 12987_2025_642_MOESM1_ESM.docx]

**Supplementary Material**

**Table S1.** Associations of choroid plexus volume with three domains of physical function in the total sample

| **Choroid plexus volume** | **β coefficient (95% confidence interval), physical function test score** | | |
| --- | --- | --- | --- |
|  | **Model 1** | **Model 2** |  |
| **SPPB balance score** (n=1169) | |  |  |
| ChP volume, continuous | -0.09(-0.15, -0.04) ^**^ | -0.08(-0.13, -0.03) ^**^ | |
| ChP volume, sex-specific quartiles^†^ | | |  |
| Q1 (n=288) | 0.00(reference) | 0.00(reference) |  |
| Q2 (n=294) | -0.13(-0.26, -0.01) ^*^ | -0.12(-0.25, 0.01) | |
| Q3 (n=287) | -0.06(-0.20, 0.07) | -0.06(-0.19, 0.08) | |
| Q4 (n=300) | -0.24(-0.38, -0.10) ^**^ | -0.20(-0.34, -0.06) ^**^ | |
| P for linear trend | 0.006 | 0.017 |  |
| **SPPB Chair stand score** (n=1216) | |  |  |
| ChP volume, continuous | -0.10(-0.17, -0.03) ^**^ | -0.09(-0.16, -0.02) ^*^ | |
| ChP volume, sex-specific quartiles^†^ | | |  |
| Q1 (n=304) | 0.00(reference) | 0.00(reference) |  |
| Q2 (n=304) | -0.08(-0.26, 0.10) | -0.08(-0.26, 0.11) | |
| Q3 (n=303) | -0.12(-0.30, 0.07) | -0.11(-0.30, 0.08) | |
| Q4 (n=305) | -0.24(-0.43, -0.05) ^*^ | -0.21(-0.41, -0.01) ^*^ | |
| P for linear trend | 0.017 | 0.041 |  |
| **SPPB Walk test score** (n=1212) | |  |  |
| ChP volume, continuous | -0.06(-0.11, -0.02) ^**^ | -0.05(-0.09, -0.01) ^*^ | |
| ChP volume, sex-specific quartiles^†^ | | |  |
| Q1 (n=304) | 0.00(reference) | 0.00(reference) |  |
| Q2 (n=304) | -0.01(-0.11, 0.11) | 0.01(-0.10, 0.13) |  |
| Q3 (n=300) | -0.05(-0.17, 0.06) | -0.04(-0.15, 0.08) | |
| Q4 (n=304) | -0.11(-0.24, 0.01) | -0.07(-019, 0.05) | |
| P for linear trend | 0.04 | 0.172 |  |

Abbreviations: ChP, choroid plexus volume; SPPB, Short Physical Performance Battery.

Note: Model 1 was controlled for age, sex, education, and total intracranial volume; model 2 was additionally adjusted for smoking, alcohol intake, body mass index, physical exercise, hypertension, diabetes, dyslipidemia, *APOE* genotype, stroke, and disproportionately enlarged subarachnoid-space hydrocephalus.

^†^The cut-offs of quartiles for total choroid plexus volume in females were <2.68 ml (Q1), 2.68-3.30 ml (Q2), 3.30-3.87 ml (Q3), and >3.87 ml (Q4) and the corresponding cut-offs in males were <3.72 ml (Q1), 3.72-4.49 ml (Q2), 4.49-5.10 ml (Q3), and >5.10 ml (Q4).

^*^P<0.05, ^**^P<0.01.

**Table S2.** Associations of choroid plexus volume with physical function in participants free of dementia and Parkinson’s disease

| **Choroid plexus volume** | **β coefficient (95% confidence interval), physical function test score** | |
| --- | --- | --- |
|  | **Model 1** | **Model 2** |
| **SPPB balance score** (n=1138) |  |  |
| ChP volume, continuous | -0.07(-0.12, -0.02) ^**^ | -0.07(-0.12, -0.02) ^**^ |
| ChP volume, sex-specific quartiles^†^ | |  |
| Q1 (n=279) | 0.00(reference) | 0.00(reference) |
| Q2 (n=286) | -0.13(-0.25, -0.01) ^*^ | -0.10(-0.22, 0.02) |
| Q3 (n=281) | -0.03(-0.15, 0.10) | -0.02(-0.15, 0.11) |
| Q4 (n=292) | -0.20(-0.33, -0.06) ^**^ | -0.16(-0.30, -0.03) ^*^ |
| P for linear trend | 0.028 | 0.067 |
| **SPPB chair stand score** (n=1185) |  |  |
| ChP volume, continuous | -0.08(-0.15, -0.01) ^**^ | -0.07(-0.14, 0.01) |
| ChP volume, sex-specific quartiles^†^ | |  |
| Q1 (n=295) | 0.00(reference) | 0.00(reference) |
| Q2 (n=296) | -0.02(-0.20, 0.16) | -0.03(-0.21, 0.16) |
| Q3 (n=297) | -0.10(-0.29, 0.09) | -0.07(-0.26, 0.12) |
| Q4 (n=297) | -0.19(-0.39, 0.01) | -0.16(-0.36, 0.04) |
| P for linear trend | 0.04 | 0.108 |
| **SPPB walk test score** (n=1181) |  |  |
| ChP volume, continuous | -0.05(-0.10, -0.01) ^**^ | -0.03(-0.08, 0.01) |
| ChP volume, sex-specific quartiles^†^ | |  |
| Q1 (n=295) | 0.00(reference) | 0.00(reference) |
| Q2 (n=296) | 0.01(-0.11, 0.11) | 0.02(-0.09, 0.13) |
| Q3 (n=294) | -0.03(-0.15, 0.08) | -0.01(-0.12, 0.10) |
| Q4 (n=296) | -0.09(-0.21, 0.03) | -0.04(-0.16, 0.08) |
| P for linear trend | 0.13 | 0.431 |
| **SPPB summary score** (n=1139) |  |  |
| ChP volume, continuous | -0.24(-0.37, -0.11) ^**^ | -0.19(-0.32, -0.07) ^**^ |
| ChP volume, sex-specific quartiles^†^ | |  |
| Q1 (n=279) | 0.00(reference) | 0.00(reference) |
| Q2 (n=287) | -0.24(-0.57, 0.08) | -0.20(-0.51, 0.12) |
| Q3 (n=282) | -0.22(-0.55, 0.11) | -0.16(-0.49, 0.16) |
| Q4 (n=291) | -0.53(-0.88, -0.19) ^**^ | -0.40(-0.74, -0.05) ^*^ |
| P for linear trend | 0.005 | 0.040 |

Abbreviations: ChP, choroid plexus volume; SPPB, Short Physical Performance Battery.

Note: Model 1 was controlled for age, sex, education, and total intracranial volume; model 2 was additionally adjusted for smoking, alcohol intake, body mass index, physical exercise, hypertension, diabetes, dyslipidemia, *APOE* genotype, stroke, and disproportionately enlarged subarachnoid-space hydrocephalus.

^†^The cut-offs of quartiles for total choroid plexus volume in females were <2.67 ml (Q1), 2.67-3.28 ml (Q2), 3.28-3.85 ml (Q3), and >3.85 ml (Q4) and the corresponding cut-offs in males were <3.71 ml (Q1), 3.71-4.50 ml (Q2), 4.50-5.10 ml (Q3), and >5.10 ml (Q4).

^*^P<0.05, ^**^P<0.01.

**Table S3** Associations of choroid plexus volume with physical function in participants who were scanned at Southwestern Lu Hospital

| **Choroid plexus volume** | **β coefficient (95% confidence interval), physical function test score** | |
| --- | --- | --- |
|  | **Model 1** | **Model 2** |
| SPPB balance score (n=1077) |  |  |
| ChP volume, continuous | -0.09(-0.15, -0.03) ^**^ | -0.07(-0.13, -0.01) ^*^ |
| ChP volume, sex-specific quartiles^†^ | |  |
| Q1 (n=264) | 0.00(reference) | 0.00(reference) |
| Q2 (n=273) | -0.08(-0.22, 0.06) | -0.10(-0.24, 0.05) |
| Q3 (n=264) | -0.02(-0.17, 0.12) | -0.03(-0.18, 0.11) |
| Q4 (n=276) | -0.17(-0.32, -0.02) ^*^ | -0.15(-0.31, 0.01) |
| P for linear trend | 0.062 | 0.111 |
| SPPB Chair stand score (n=1119) |  |  |
| ChP volume, continuous | -0.09(-0.17, -0.01) ^**^ | -0.07(-0.15, 0.01) |
| ChP volume, sex-specific quartiles^†^ | |  |
| Q1 (n=279) | 0.00(reference) | 0.00(reference) |
| Q2 (n=280) | -0.08(-0.27, 0.11) | -0.02(-0.22, 0.18) |
| Q3 (n=280) | -0.12(-0.31, 0.08) | -0.07(-0.27, 0.13) |
| Q4 (n=280) | -0.26(-0.47, -0.06) ^*^ | -0.16(-0.37, 0.05) |
| P for linear trend | 0.012 | 0.114 |
| SPPB Walk test score (n=1116) |  |  |
| ChP volume, continuous | -0.05(-0.10, -0.01) ^**^ | -0.03(-0.08, 0.02) |
| ChP volume, sex-specific quartiles^†^ | |  |
| Q1 (n=280) | 0.00(reference) | 0.00(reference) |
| Q2 (n=280) | 0.01(-0.11, 0.13) | 0.05(-0.07, 0.17) |
| Q3 (n=277) | -0.02(-0.14, 0.10) | -0.01(-0.13, 0.12) |
| Q4 (n=279) | -0.10(-0.23, 0.03) | -0.03(-0.15, 0.11) |
| P for linear trend | 0.109 | 0.521 |
| SPPB Summary score (n=1078) |  |  |
| ChP volume, continuous | -0.26(-0.41, -0.13) ^**^ | -0.20(-0.34, -0.05) ^**^ |
| ChP volume, sex-specific quartiles^†^ | |  |
| Q1 (n=264) | 0.00(reference) | 0.00(reference) |
| Q2 (n=274) | -0.25(-0.60, 0.09) | -0.18(-0.53, 0.19) |
| Q3 (n=265) | -0.22(-0.58, 0.13) | -0.17(-0.54, 0.19) |
| Q4 (n=275) | -0.59(-0.96, -0.22) ^**^ | -0.39(-0.77, -0.01) ^*^ |
| P for linear trend | 0.004 | 0.062 |

Abbreviations: ChP, choroid plexus volume; SPPB, Short Physical Performance Battery.

Note: Model 1 was controlled for age, sex, education, and total intracranial volume; model 2 was additionally adjusted for smoking, alcohol intake, body mass index, physical exercise, hypertension, diabetes, dyslipidemia, *APOE* genotype, stroke, and disproportionately enlarged subarachnoid-space hydrocephalus.

^†^The cut-offs of quartiles for total choroid plexus volume in females were <2.81 ml (Q1), 2.81-3.35 ml (Q2), 3.35-3.93 ml (Q3), and >3.93 ml (Q4) and the corresponding cut-offs in males were <3.88 ml (Q1), 3.88-4.57 ml (Q2), 4.57-5.16 ml (Q3), and >5.16 ml (Q4).

^*^P<0.05, ^**^P<0.01.
